# Effects of Cigarette Smoke on TSPO-related Mitochondrial Processes

**DOI:** 10.3390/cells8070694

**Published:** 2019-07-10

**Authors:** Nidal Zeineh, Rafael Nagler, Martin Gabay, Abraham Weizman, Moshe Gavish

**Affiliations:** 1 The Ruth and Bruce Rappaport Faculty of Medicine, Technion Institute of Technology, Haifa 31096, Israel; 2 Research Unit at Geha Mental Health Center and Laboratory of Biological Psychiatry at Felsenstein Medical Research Center, Petah Tikva 4910002, Israel; 3 Sackler Faculty of Medicine, Tel Aviv University, Tel Aviv 6997801, Israel

**Keywords:** TSPO, cAMP, cigarette smoke, mitochondrial membrane potential, ROS, apoptosis, necrosis, cell death

## Abstract

The 18 kDa translocator protein (TSPO) is an initiator of the mitochondrial apoptosis cascade. Cigarette smoke (CS) exposure provokes alterations in TSPO expression as well as upregulation of its related functions such as mitochondrial membrane potential (Δψ_M_) and reactive oxygen species generation, which are associated with cell death. In the current study, H1299 lung cancer cell line exposed to CS for various time periods (30 mins, 60 mins and 120 mins) and TSPO expression and cell death processes were studied. CS exposure for 30 mins resulted in a non-significant increase in TSPO expression by 24% (*p* > 0.05 vs. control). CS exposure for 60 mins and 120 mins resulted in a significant increase by 43% (*p* < 0.05 vs. control) and by 47% (*p* < 0.01 vs. control), respectively. Furthermore, TSPO-related mitochondrial functions were upregulated at the 120 mins time point following CS exposure. TSPO expression is upregulated by CS, suggesting that TSPO plays a role in cell death processes induced by CS exposure. Alterations in TSPO-related cell death processes suggest that TSPO may be involved in the tissue damage caused by CS.

## 1. Introduction

Tobacco smoke, particularly cigarette smoke (CS), is a major concern regarding human health, and is considered to be the second most common cause of death worldwide, which is rapidly expanding, imposing a worldwide menace to human health [1,2,3]. CS prevalence has slightly decreased among men and women from 39% to 35% and from 8% to 6%, respectively, during the years 2007 to 2015 [4]. CS contains approximately 250 toxic compounds [5], and causes approximately 6 million death cases per year worldwide. Furthermore, it is predicted that tobacco use will lead to more than 8 million death cases per year by the year 2030 [1]. In addition, the life span of smokers is approximately 10 years shorter than in non-smokers [6]. CS appears to affect various organ systems, and mainly leads to diseases of the respiratory tract such as chronic obstructive pulmonary diseases (COPD) and cancer, particularly lung, laryngeal and tongue cancers [7,8]. Lungs are the primary target of CS injury due to a direct chemical insult. On the other hand, indirect consequences result in chronic diseases in distant organ systems [9]. The mechanisms responsible for the CS-induced tissue damage are unclear.

The 18 kDa Translocator protein (TSPO) is a cholesterol binding protein that can be upregulated in numerous neoplastic and inflammatory disorders [10,11,12,13,14]. TSPO is highly expressed in steroid- synthesizing cells of endocrine organs [15,16]. Inside the cell, it is mainly located on the outer mitochondrial membrane (OMM) [15,17]. TSPO is involved in several functions such as programmed cell death, inflammation, cancer, oxidative stress, and mitochondrial membrane potential regulation [15,17,18,19,20,21,22,23,24]. Moreover, the role of TSPO in the regulation of mitochondrial membrane potential (Δψ_M_) collapse is relevant to apoptotic cell death process [24,25,26,27]. Lethal agents can lead to activation of TSPO, and subsequent ROS generation and mitochondrial membrane potential collapse that eventually leads to cell death [25]. TSPO alterations lead to modification of numerous mitochondrial processes, including the ATP synthase activity in the inner mitochondrial membrane. Hence, downstream changes are reflected in ROS and cardiolipin peroxidation levels [28]. Tracking of ADP/ATP ratio can be indicative of various biological processes and severe pathologies [29,30]. ATP plays a major role in energy storage inside the cell [31] and decreased ATP levels and increased ADP levels indicate apoptotic and necrotic processes. Also, it is known that elevated cardiolipin peroxidation leads to release of apoptogenic proteins, such as cytochrome c, from the mitochondria into the cytosol. In the cytosol, along with adaptor protein, apoptotic protease activating factor (Apaf-1) and caspase-9 form apoptosomes. Eventually, these complexes subsequently lead to apoptosis [28]. In addition to the various functions of the TSPO, it is in close relation to cyclic adenosine monophosphate (cAMP), which is a well-studied second messenger that is involved in the regulation of cell death [32]. Certain components of the cAMP signaling pathway can be targeted in the treatment of different cancers, in an attempt to enhance apoptotic levels [33,34]. An increase in cAMP can have a pro-apoptotic effect in numerous cell types including the H1299 lung cancer cell line, while in other cell types, such as neuronal cell types and keratinocytes, cAMP can play an anti-apoptotic role, including the blockade of spontaneous apoptosis and apoptosis induced by a variety of agents [32]. 

Since TSPO is involved in metabolic pathways relevant to cell death, inflammation and mitochondrial activities, we hypothesized that the toxic effects of CS on human lung cancer cell line are dependent on alterations in the TSPO expression. 

In the current study, we focused on CS-induced cellular damages including alterations in ATP levels, ROS generation, cardiolipin peroxidation, Δψ_M_ collapse, cAMP, and cell death, after exposure of lung cancer cell line H1299 cells to CS for 30 mins, 60 mins, and 120 mins.

## 2. Materials and Methods

### 2.1. Study Design

Lung cancer cell line cells (H1299) from human origin were used in this study. The cells were maintained as described by the American Type Culture Collection (ATCC, Manassas, VA, USA). Full culture medium consisted of RPMI (high glucose, with no l-Glutamine and no sodium pyruvate), supplemented with fetal bovine serum (FBS; 10%), glutamine (2%) and gentamycin (50 mg/mL). The cells were incubated in this full culture medium for 48 hours, in petri dishes or well plates as required, at 37 °C and 5% CO_2_ until approximately 80% confluency was reached, then incubated in serum deprived (0.5% FBS) medium (starvation medium; in order to enhance the sensitivity of the cells to CS) with 1% ethanol (vehicle) for 24 hours. 

Following the incubation procedure in starvation medium with 1% ethanol (vehicle), the cells were exposed for 30 mins, 60 mins, and 120 mins to CS, as described previously [35,36,37]. The experimental groups included a control group with starvation medium contining1% ethanol (vehicle) and exposed to fresh air; and a CS group containing cells grown in starvation medium with 1% ethanol (vehicle) and exposed to CS for 30 mins, 60 mins and 120 mins. 

### 2.2. Exposure of H1299 Cells to Cigarette Smoke

Prior to CS exposure, the H1299 cells were seeded and incubated with full culture medium for 48 hours in wells or plates, as appropriate, to reach the desired confluency (approx. 80% confluency) for the experiments. This was followed by incubation for 24 hours in starvation medium, and then 30 mins, 60 mins, and 120 mins of CS exposure.

For CS exposure, the cell cultures were placed in a vacuum sealed chamber with a tube attached holding the burning cigarette. The cigarettes used were filtered “Time” cigarettes (Dubek, Petah Tikva, Israel) containing 14 mg of tar and 0.9 mg of nicotine per cigarette. CS was drawn in by using a lowered pressure system [35,36,37]. Briefly, a vacuum pump was utilized in order to create a low pressure (0.5–0.6 bar) inside the chamber and then a valve was closed. The cigarette was lit, and simultaneously under pressure, another valve was opened in order to suck the smoke into the sealed chamber. After the entire cigarette was burned, this valve was closed and the chamber containing the smoke and the experimental plates remained sealed for 15 minutes. Every 15 minutes, a new cigarette was lit. Control groups were placed in a chamber exposed to fresh air [38,39].

### 2.3. The Effect of CS Exposure on TSPO and cAMP Levels

Cells were trypsinized and collected together with medium after being exposed to CS or fresh air. The cells were centrifuged (660× *g* for 5 min) and the medium was removed. The cells were fixed in Paraformaldehyde (4% in DDW) for 10 min then washed with PBS without Ca^+2^ and Mg^+2^. The cells were resuspended in 800 μL of PBS containing 0.2% Tween (PBS-T) for 10 mins on ice. The cells were washed again with PBS and resuspended in 100 μL of PBS-T containing 3% BSA and anti-TSPO or anti-cAMP antibodies (1:100) (Abcam, Cambridge, UK). The resuspended samples were incubated overnight at 4 °C. On the next day, the cells were washed and resuspended in 100 μL of PBS-T containing 3% BSA and Alexa Fluor 488 AffiniPure Goat Anti-Rabbit IgG (1:1.000) (Abcam, Cambridge, UK). Mean fluorescence intensity (MFI) was measured using fluorescence assisted cell sorting (FACS) with a CyAN ADP FACS machine (Beckman Coulter, Brea, CA, USA). The data were analyzed using FlowJo software (10th version, FlowJo LLC, Ashland, OR, USA).

### 2.4. ADP/ATP Ratio

Following exposure of the CS group and control group to CS or fresh air, respectively, ATP and ADP levels were measured using ELISA. According to the manufacturer protocol, ADP/ATP ratio assay was performed using a commercial assay kit (MAK135; Sigma-Aldrich, St. Louis, MO, USA), as previously described [40]. Luminescence was measured using Infinite M200 Pro plate reader (Tecan, Männedorf, Switzerland). ADP/ATP ratios were calculated as follows:

$$\frac{\mathrm{ADP}}{\mathrm{ATP}}ratio=\frac{RLU_{C}\;\left(ADP\;signal\right)-RLU_{B}\;\left(Background\;signal\right)\;}{RLU_{A}\;\left(ATP\;signal\right)}$$

RLU = *Luminescence Reading*

### 2.5. Cardiolipin Peroxidation Levels

Cardiolipin is a mitochondrial specific phospholipid, located in the inner mitochondrial membrane. Dissociation of cytochrome c from the inner mitochondrial membrane, for example, due to peroxidation of cardiolipins, is an initial step in the activation of the mitochondrial apoptotic cascade [25]. Cells were trypsinized and collected by centrifugation (660× *g*, 5 mins, 4 °C). This was followed by the resuspension of the sample in 400 µL of the fluorescent dye 10-*N*-Nonyl-Acridine Orange (NAO) (1/1000 dilution in PBS) and incubation in the dark for 30 mins. Then, the samples were centrifuged, resuspended in 500 µL of PBS and transferred into FACS tubes, as described previously [41]. The MFI of NAO labeling exhibited negative linear correlation to cardiolipin peroxidation levels, as was assessed by FACS [42]. The results were analyzed using FlowJo (10th version, FlowJo LLC, Ashland, OR, USA).

### 2.6. Collapse of the Mitochondrial Membrane Potential (ΔΨm)

Collapse of the ΔΨm can lead to the initiation of the mitochondrial apoptotic cascade. The cationic lipophilic JC-1 (5,5′,6,6′-tetrachloro-1,1′,3,3′-tetraethylbenzimidazolylcarbocyanine-chloride) dye was utilized as an indicator of changes in ΔΨm, as previously described [18]. In intact cells with high ΔΨm, JC-1 can enter the mitochondria and reversibly form aggregates with intense red fluorescence (emission at 590 nm; orange-red fluorescence). In case of ΔΨm collapse, JC-1 remains in the cytosol as monomers and emits at 527 nm (green fluorescence). Cells were trypsinized, centrifuged (660× *g*, 5 mins, 4 °C), and collected. For the positive control group, the proton ionophore carbonyl cyanide m-chlorophenylhydrazone (CCCP) was used as described previously [18]. Cells were resuspended and incubated with 400 µL of JC-1 dye (1/500 dilution) for 30 mins. Then, the cells were centrifuged, resuspended in 500 µL of PBS and transferred into FACS tubes. The MFI of JC-1 labeling was measured by FACS. The results were calculated using FlowJo (10th version, FlowJo LLC, Ashland, OR, USA).

### 2.7. Cellular Cytotoxicity Measurement by LDH Enzyme Activity 

Total cellular cytotoxicity as measured by LDH enzyme levels in the medium surrounding the cells. In case of necrosis and late apoptosis, LDH is released from the cells when the cell membrane is compromised [43]. Briefly, Cytotoxicity Detection Kit (LDH) (Roche pharmaceuticals, Basel, Switzerland) was applied according to the manufacturer’s protocol (absorbance at 492 nm wavelength with reference wavelength of 620 nm). Measurements were performed with a spectrophotometer Zenyth 200 (Anthos, Eugendorf, Austria) and the results were calculated and normalized according to the formula given by the manufacturer.

### 2.8. Apoptosis and Necrosis Levels as Measured by FACS

For the detection of the levels of apoptosis and necrosis following CS exposure for different time periods, we used the Apoptosis/Necrosis detection kit (Abcam, Cambridge, UK), according to the manufacturer’s instructions. The cells were trypsinized, centrifuged (660× *g*, 5 mins, 4 °C), and collected. For every sample, 200 µL of assay buffer was added followed by the addition of: 2 μL Apopxin Green Indicator (200X), 1 μL 7-AAD (200X), and 1 μL CytoCalcein violet 450 (200X) to detect apoptosis, necrosis, and intact cells, respectively. After 60 mins of incubation, fluorescence was measured using Aria FACS machine (BD bioscience, San Jose, CA, USA) and the results were calculated by FlowJo (10th version, FlowJo LLC, Ashland, OR, USA).

### 2.9. Statistical Analysis

The program used for statistical analysis was GraphPad Prism (5th version, GraphPad Software, San Diego, CA, USA). Data were expressed as mean ± SEM. Two-way ANOVA followed by Bonferroni’s post hoc test for multiple comparisons were performed as appropriate and *p* < 0.05 was considered as statistically significant.

## 3. Results

### 3.1. TSPO Levels

**Figure 1:**
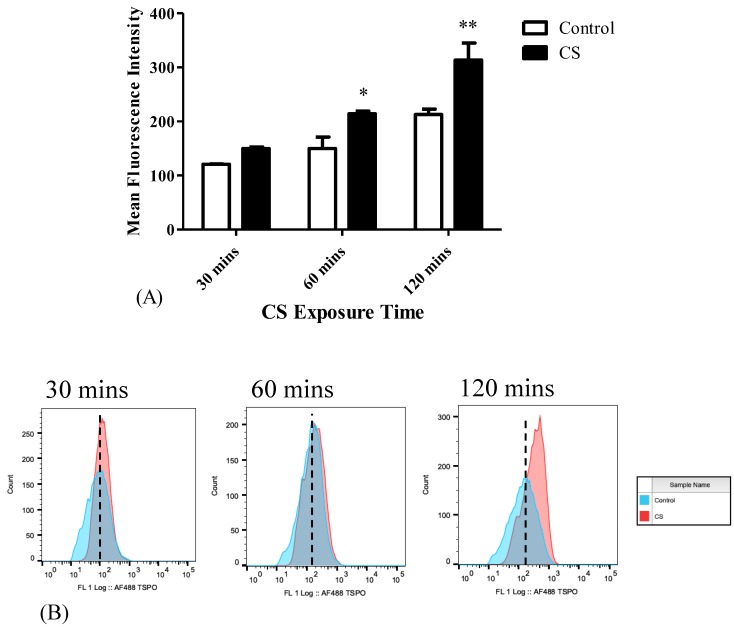
The impact of CS exposure on TSPO levels in H1299 cells. Mean Fluorescence Intensity (MFI) was calculated using FACS to assess the TSPO levels following CS exposure. (**A**) Bar graph of MFI, and (**B**) Corresponding histograms. The results are expressed as MFI ± SEM (n = 4 in each group). Two-way ANOVA followed by Bonferroni post hoc test was performed. * *p* < 0.05 and ** *p* < 0.01 compared to the corresponding control group. 30 mins Control vs. 120 mins Control (*p* < 0.05).

Levels of TSPO expression were measured using FACS at different CS exposure times. Initially, following 30 mins of CS exposure, TSPO expression in the CS group increased by 24%, as compared to the control group, without statistical significance. However, after 60 mins and 120 mins of CS exposure, significant increases in TSPO levels by 43% (*p* < 0.05) and by 47% (*p* < 0.01), respectively, were observed in the CS group as compared to the control group (Figure 1). The corresponding histogram analysis of the distribution of the fluorescence signals in the population of labeled cells are depicted in Figure 1B.

### 3.2. ADP/ATP Ratio

**Figure 2:**
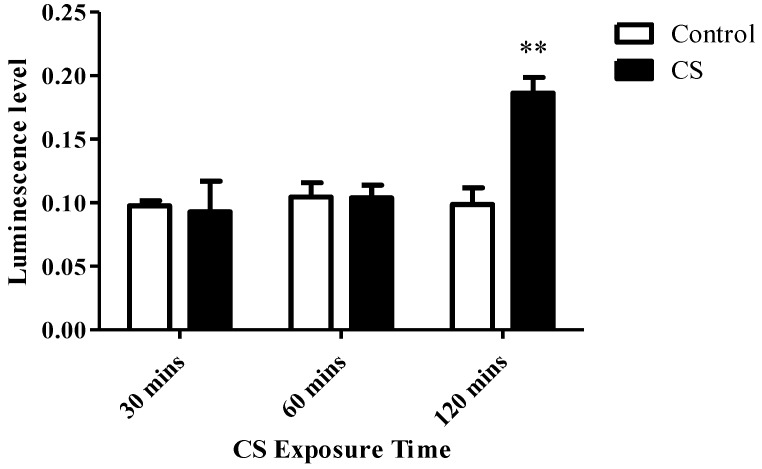
The effects of CS exposure on ADP/ATP ratio. ADP/ATP ratio was calculated using ELISA following CS exposure for 30, 60, and 120 mins. The results are expressed as mean luminescence intensity ± SEM (n = 4 in each group). Two-way ANOVA followed by Bonferroni post hoc test was performed. ** *p* < 0.01 as compared to the corresponding control group.

Following 30 mins and 60 mins of CS exposure, no significant alterations in ADP/ATP ratio were observed as compared to the control group, while after 120 mins of CS exposure, a significant increase by 88% (*p* < 0.01) was obtained as compared to the control group (Figure 2). 

### 3.3. Cardiolipin Levels 

**Figure 3:**
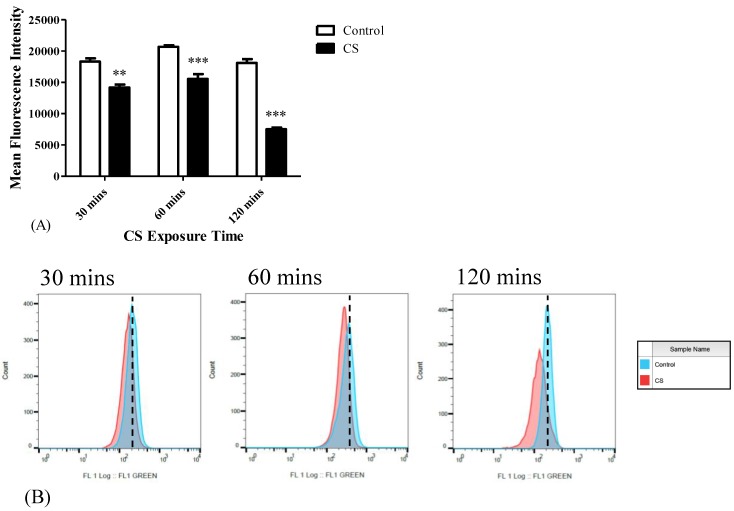
The effects of CS exposure on cardiolipin levels. Mean Fluorescence Intensity (MFI) was calculated using FACS to assess the cardiolipin content following CS exposure. (**A**) Bar graph of MFI, and (**B**) Corresponding histograms. The results are expressed as MFI ± SEM (n = 4 in each group). Two-way ANOVA followed by Bonferroni post hoc test was performed. ** *p* < 0.01 and *** *p* < 0.001 as compared to corresponding control groups.

Cardiolipin levels were also measured following different CS exposure times (30 mins, 60 mins, 120 mins). Significant decreases in MFI of NAO by 22% (*p* < 0.01), by 25% (*p* < 0.001), and by 59% (*p* < 0.001) were detected following 30 mins, 60 mins, and 120 mins of CS exposure, respectively, as compared to the corresponding control groups (Figure 3A). The corresponding histogram analysis of the distribution of the fluorescence signals in the population of labeled cells are depicted in Figure 3B.

### 3.4. Collapse of the Mitochondrial Membrane Potential (ΔΨm) 

**Figure 4:**
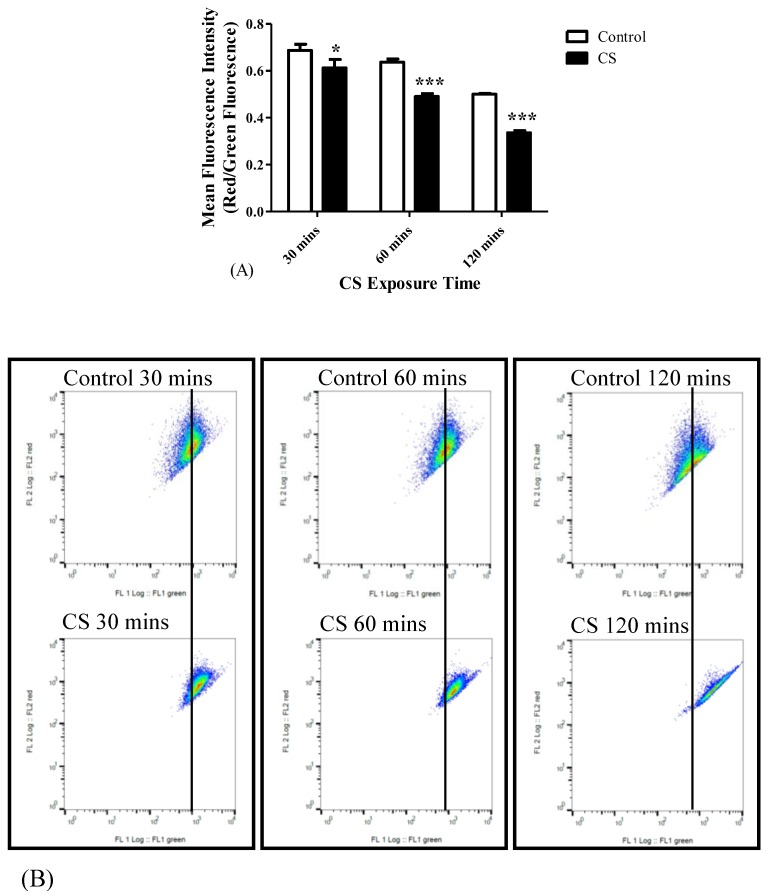
The impact of CS exposure on the mitochondrial membrane potential collapse. After exposure to 30 mins, 60 mins, and 120 mins to CS, the ratio between red and green Mean Fluorescence Intensity (MFI) was determined using FACS to assess the mitochondrial membrane potential. (**A**) Bar graph of MFI, and (**B**) Corresponding Red/Green ratio histograms. Results are expressed as the ratio of Red/Green fluorescence intensity as mean ± SEM (n = 4 in each group). Two-way ANOVA followed by Bonferroni post hoc test was performed. * *p* < 0.05 and *** *p* < 0.001 compared to the corresponding control groups. 30 mins Control vs. 120 mins Control (*p* < 0.001) and 60 mins Control vs. 120 mins Control (*p* < 0.01).

As mentioned before, the Δψ_M_ was measured using the lipophilic JC-1 dye. Δψ_M_ collapse was observed following all three time points of CS exposure. The changes in the red to green fluorescence ratios in the tested cell populations following different CS exposure times are depicted in Figure 4. As shown, a decrease of the ratio of red/green fluorescence by 11% (*p* < 0.05) occurred following 30 mins, by 23% (*p* < 0.001) occurred at 60 mins, and by 33% (*p* < 0.001) occurred at 120 mins of CS exposure, as compared to the corresponding control groups (Figure 4A). The corresponding histogram analysis of the distribution of the fluorescence signals in the population of labeled cells are depicted in Figure 4B. 

### 3.5. Cellular Cytotoxicity (LDH)

**Figure 5:**
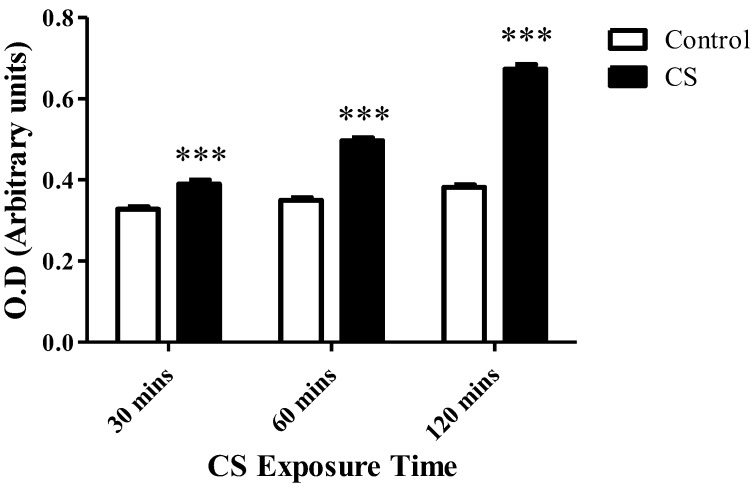
The impact of CS on cell death. LDH enzyme levels released to the surrounding medium were assessed using LDH cytotoxicity assay kit, following CS exposure time periods of 30 mins, 60 mins, and 120 mins, as compared to the corresponding control groups. The results are expressed as mean ± SEM (n = 4 in each group) of cytotoxicity level as measured by O.D. (arbitrary units). *** *p* < 0.001 compared to the corresponding control groups.

Cellular cytotoxicity levels increased gradually with CS exposure time: Significant increases of 19% (*p* < 0.001), 42% (*p* < 0.001), and 76% (*p* < 0.001) in the levels of LDH in the medium were detected after 30 mins, 60 mins, and 120 mins of CS exposure, respectively, as compared to the corresponding control groups (Figure 5). 

### 3.6. Apoptosis and Necrosis Levels 

**Figure 6:**
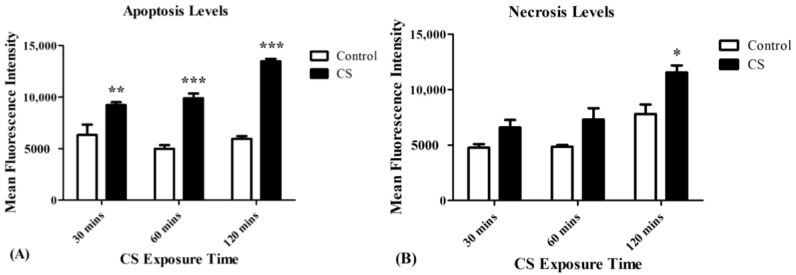
The impact of CS on apoptotic and necrosis cell death. Mean Fluorescence Intensity (MFI) was used to assess the levels of apoptotic and necrotics cell death following CS exposure. (**A**) The proportional elevation in apoptotic cell death following CS exposure during the 3 time periods of CS exposure. (**B**) Necrotic cell death levels following CS exposure of 30 mins, 60 mins, and 120 mins, compared to the corresponding control groups. Results are expressed as MFI and demonstrated as Means ± SEM (n = 4 in each group). Two-way ANOVA followed by Bonferroni post hoc test was performed. * *p* < 0.05, ** *p* < 0.01 and *** *p* < 0.001 compared to the corresponding control groups.

A linear correlation between increased apoptosis levels, as assessed by Apopxin Green Indicator (200X), and increased exposure times to CS was recorded. Apoptosis levels were significantly increased by 48% (*p* < 0.01), 98% (*p* < 0.001), and 127% (*p* < 0.001) following 30 mins, 60 mins, and 120 mins of CS exposure, respectively, as compared to the corresponding control groups (Figure 6A). Necrosis levels increased significantly in the CS group, as assessed by 7-AAD (200X) only after 120 mins of CS exposure (48%; *p* < 0.05) as compared to the corresponding control group (Figure 6B).

### 3.7. cAMP Levels

**Figure 7:**
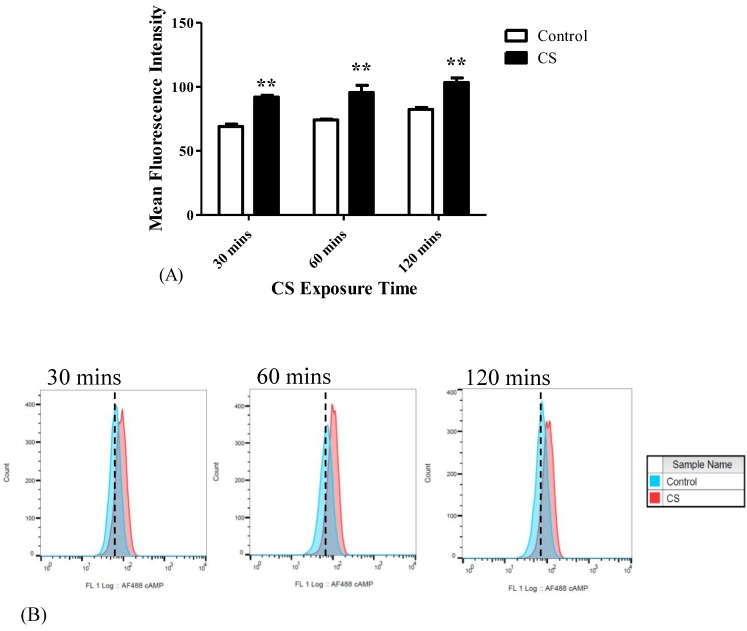
cAMP levels following CS exposure. After CS exposure for 30 mins, 60 mins, and 120 mins, the cAMP levels were assessed as a pro-apoptotic marker using FACS. (**A**) Bar graph of MFI, and (**B**) Corresponding histograms. The results are expressed as MFI ± SEM (n = 4 in each group). Two-way ANOVA followed by Bonferroni post hoc test was performed. ** *p* < 0.01 compared to the corresponding control groups.

cAMP levels were measured following CS exposure as a pro-apoptotic marker. CS exposure resulted in significant increases in the levels of cAMP by 33% (*p* < 0.01) at 30 mins, by 28% (*p* < 0.01) at 60 mins, and 25% (*p* < 0.01) at 120 mins of CS exposure, as compared to their corresponding control groups (Figure 7A). The corresponding histogram analysis of the distribution of the fluorescence signals in the population of labeled cells are depicted in Figure 7B.

## 4. Discussion

**Figure 8:**
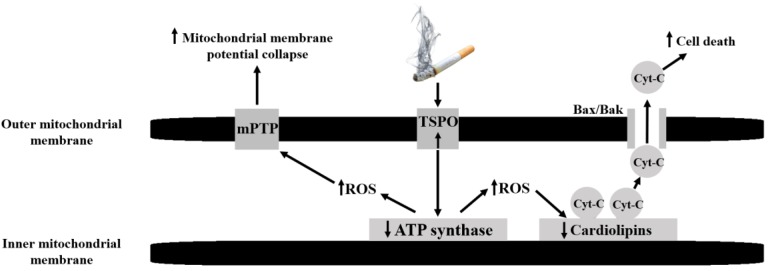
Schematic illustration representing the cascade of the cigarette smoke (CS) effect on mitochondrial components. The mitochondrial events start with an increase in TSPO expression on the outer mitochondrial membrane in response to CS, leading to a decrease in ATP synthase activity, ROS generation and cardiolipin peroxidation (a decrease in cardiolipin content) followed by collapse of Δψ_M_, which stimulates the release of cytochrome c putatively by the Bax/Bak channel, and elevation in cAMP and LDH, that eventually results in cell death (apoptosis and necrosis).

In the current study, we investigated the effects of increasing CS exposure time periods (30 mins, 60 mins, 120 mins) on TSPO levels and its related mitochondrial/cellular functions in H1299 cells. One important role of TSPO is its involvement in programmed cell death process, in addition to its other cellular functions [15,17]. TSPO participates also in the regulation of the mitochondrial membrane potential and ROS generation. Over-activation of TSPO via these processes eventually lead to cell death in human neuroblastoma cells [44]. In the current study, we demonstrated that the previous mentioned functions of TSPO are time-dependent in the context of CS exposure. Namely, cell death levels as assessed by LDH levels, were elevated by 19%, 42%, and 76% after 30 mins, 60 mins, and 120 mins of CS exposure, respectively. Similarly, alterations in other TSPO-related functions, including ATP synthase activity (ADP/ATP ratio), cardiolipin peroxidation, mitochondrial membrane potential collapse, cell death (mainly apoptosis and necrosis), and cAMP levels, all were demonstrated to be sensitive to CS exposure in a time-dependent manner. The cascade of cellular events related to CS exposure and activation of TSPO are illustrated in Figure 8. 

The relation between TSPO and ATP synthase was previously demonstrated using TSPO knockdown experiments [28]. Similar to CS, TSPO activation by synthetic ligands, is associated with reversal of the ATP synthase proton pump in the inner mitochondrial membrane, and eventually leads to oxidative stress and ROS generation to occur. In turn, this leads to cardiolipin peroxidation associated binding of cytochrome c and its release. In parallel to cardiolipin peroxidation, elevation in ROS causes modifications in the mitochondrial permeability transition pore (MPTP) and efflux of Ca^+2^ into the cytosol [24,25,26,27], followed by collapse of Δψ_M_ which causes alteration in OMM channels, such as Bax/Bak, and the release of cytochrome c into the cytosol. This cascade of events results mainly in the activation of the apoptotic cascade that includes formation of apoptosomes and eventually leading to cell death [28] (Figure 8). The underlying mechanisms by which CS exerts its cytotoxic effects remain unclear; however, it is likely that some of the compounds of the CS can stimulate the expression and/or activation of the TSPO. Additionally, it is possible that ROS, along with other DNA aberrations cause cellular damage resulting in cell death [45,46]. Moreover, CS can lead to cellular death via pathways which are not dependent on the putative activity of TSPO. Such relevant pathways may include PI3K/Akt/mTOR and the MAPK [28]. Nevertheless, CS-induced upregulation of TSPO may result in release of pro-apoptotic molecules, including cytochrome c, through Bax/Bak in the damaged OMM into the cytosol, and this ultimately initiates a process of programmed cell death [25,28,47].

On the cellular level, it appears that the effects of CS on TSPO-related functions including ATP synthase activity, ROS generation, ΔψM regulation, and cell death, are affected by CS duration. These data corroborate a previous study showing time-dependent effects of CS on cell death of HaCaT keratinocytes, in which increased amounts of CS exposure linearly correlated with increased carbonyl levels [48]. Moreover, similar patterns were demonstrated in lymphocytes, as 44% of reduction in survival was detected following 80 mins of CS exposure compared to 20% of reduction in survival after 20 mins of CS exposure [49]. Also, in the oral cancer cell lines SCC-15 and SCC-25, a time-dependent reduction in survival rates were observed following 60 mins and 120 mins of CS exposure [50].

It seems that the cytotoxic effect of CS was achieved by TSPO-related or TSPO-unrelated apoptosis since apoptosis was detected already at 30 mins and persisted for a duration of 120 mins (Figure 6A), while necrotic death was achieved only after 120 mins (Figure 6B). Furthermore, a similar pattern was observed in cardiolipin peroxidation (Figure 3), along with significant ADP/ATP ratio changes (Figure 2), which are proxy markers for apoptotic and necrotic cell death, respectively. The similarity in patterns of elevation in TSPO levels and apoptotic cell death levels, suggests a correlation between the two processes and the putative important role of TSPO in apoptotic cell death. On the other hand, the lack of such a correlation between TSPO expression and necrotic cell death pattern following CS exposure, supports further the specificity of the correlation between TSPO and apoptotic cell death, rather than with necrotic cell death. Moreover, previous studies demonstrated the proapoptotic activity of TSPO in other cell lines [18,51].

It is already known that CS exposure is able to directly activate TRPA1 channels, leading to an influx of extracellular Ca^+2^, thereby triggering several downstream events, which could be related to the current observed effects [52,53]. Further studies are needed to evaluate in depth other CS-sensitive processes along with relevant biomarkers, such as apoptotic cell death markers (e.g., bcl2), Ca^+2^-related processes affected by CS, using Ca^+2^-imaging methods, neuroinflammatory effects of CS extracts, and cAMP-related processes associated with CS exposure and tissue damage. At this stage it is unclear whether cell death or apoptosis after CS either dependent, independent, or both on TSPO. Future knockout studies are needed to clarify this issue.

In summary, our current results show that TSPO and its related mitochondrial functions are sensitive to CS. Exposure of H1299 cells to CS is associated with upregulation of TSPO expression and initiation of a cascade of cellular events associated with cell death. Such alterations may be relevant to pathological effects of CS and the impact of TSPO and TSPO-related mitochondrial functions in the context of CS exposure. The TSPO-related effects may be particularly relevant to pulmonary diseases associated with CS, such as inflammation and cancer. Our results may have implications for the mechanisms via which CS exerts its lethal effects on pulmonary cells. 

Moreover, if lung cancer cells are more sensitive than healthy lung cells to the pro-apoptotic and pro-necrotic effects of TSPO ligands, such ligands may serve as potential candidates for the treatment of lung cancer. Such a therapeutic option merits further in vitro and in vivo investigations.
